# Notes and News

**Published:** 1890-01-04

**Authors:** 


### Notes and News.

Collected and Communicated.

A loan exhibition of pictures and other works of art in aid of the building fund of the Great Northern Central Hospital is to be opened by her Royal Highness Princess Louise (Marchioness of Lorne) on January 6. The exhibition will be held in the gallery of the Camden School of Art, St. Bartholomew-road, Camden-road. The exhibition will consist of works by Sir F. Leighton, Sir J. Millais, M. Alma Tadema, and other well-known artists.

At the quarterly board of Bristol Infirmary the president gave some forecast of the forthcoming annual report. He stated that the legacies received during 1889 had been more numerous than usual, but the infirmary still needed the steady support of annual subscribers. The Nurses' Home was finished and furnished, and would shortly serve as an institute from which nurses could be had for private cases. The revision of the rules, in which Dr. Shekleton has taken chief part, was referred to, and they were unanimously adopted.

The annual meeting of subscribers to the Robertson Stewart Hospital, at Rothesay, was rather disappointing. There is a strong feeling on the island in favour of a cottage hospital, so that the treatment of non-infectious cases could be carried on in a separate building. The committee had to announce that they had found no suitable site, and had not promised pecuniary support to enable them to carry out this idea. The report of the present hospital showed the income, including a balance brought forward, to be £137 11s. 4d., and the expenditure £57 3s. 4d., leaving a balance in hand of £80 8s., being an increase of about £22.

The Archbishop of Dublin presided at the annual meeting of subscribers to Jervis-street Hospital, and delivered a forcible speech on the building of the new hospital, and on the Dublin hospital grants. Large grants of public money, amounting to over £16,000 per annum, have been paid for many years by the State in support of certain hospitals in the city. Many of these hospitals so subsidised have been partly built by State funds. No portion of this £16,000 has been allotted to Jervis-street Hospital. A Bill to remove this strange anomaly was nearly passed by Parliament last session, and it is to be hoped that early in the coming session such an injustice will be remedied. One of the new wards cannot be used for lack of funds. The treasurer acknowledged a number of subscriptions, including £105 from his Grace the Archbishop, £100 from Mr. Charles Brennan (Phœnix Brewery), £100 from Mr. Charles Kennedy, J.P., and £100 from Messrs. John Power and Sons. Altogether, over £600 was promised at the meeting.

Norfolk and Norwich Hospital Sunday Fund shows a considerable falling off this year, and amounts to less than that raised in any of the past six years. It has been divided as follows: Norfolk and Norwich Hospital, £544; Jenny Lind Infirmary, £80; Norwich Dispensary, £36; Norwich Lying-in Charity, £24; Norwich Eye Infirmary, £28; Norwich Homœopathic Dispensary, £8; Norfolk and Norwich Staff of Nurses, £16; Lowestoft Convalescent Home, £40; Children's Convalescent Home, Yarmouth, £24; Surgical Appliance Society, £2; balance in hand, £3; total, £805.

Subscriptions to the amount of £229 have been received towards a dispensary building for Chorley, and a public meeting has been held to consider the subject. The attendance was small, and it did not seem as if the public took much interest in the subject. Mr. Goulding moved "That this meeting approves of the provision of a dispensary house for Chorley, and suggests £1,000 as the probable cost of a suitable building." Alderman Heald seconded the motion, which was carried unanimously. The site for the building is already secured by the generosity of the Rev. J. J. Lennon.

During December a meeting was held of medical men practising in the North of England, chiefly in Manchester, Liverpool, and Leeds, at the Queen's Hotel, Manchester, to consider the desirableness of forming an obstetrical and gynæcological society for the Northern counties. Prof. John Wallace (Liverpool) was in the chair, and among those present were Drs. Sinclair, Westmorland, Lauder, Carr, Helm, and Gilmour, of Manchester; Drs. Rumboll and Braithwaite of Leeds, Dr. Garner, of Preston, and Drs. Gemmell, Smart, Brannigan, Davies, Grimsdale, Rentoul, Burton, and Alexander, of Liverpool. On the proposition of Dr. Braithwaite, seconded by Dr. Burton, it was unanimously resolved, after discussion, that it is desirable to establish an obstetrical and gynæcological society for the North of England, with centres in Manchester, Liverpool, and Leeds. A provisional committee was appointed to carry the resolution into effect.

The *Philadelphia Public Ledger* has the following: "Next Tuesday will be the Donation Day of 'The Sheltering Arms.' This excellent charity admits within its walls infants deserted by their mothers, women deserted by their husbands, and young unmarried women with their infants, homeless but for the protection offered them by the institution. It was founded by the late Bishop Stevens, who took a deep interest in it as long as he lived, and is now presided over by the Right Rev. O. W. Whitaker. About 300 souls were cared for last year at the cost of 4,000dols. It was among the brave things that Bishop Stevens is remembered by, to found this institution, when some harsh-constructed Christians would have gone by on the other side from all such sorrowful and forlorn claimants. Now it is an established means of good—a saving institution keeping mother and child together, and helping them to make a clean way in the world again. There are peculiar landmarks which show the rising ground of Christian civilisation, and such a helpful home as the Sheltering Arms was founded to be stands on the heights, indeed, and not on lower ground. It ought to be bountifully remembered always, and especially now, as Mrs. Harrison reminds us, as it only comes before the public to ask once a year." The resident physician is an English lady, Dr. Charlotte Abbey, who, after devoting some years to the management of afflicted children in this country, notably in the Children's Hospital at Birmingham, took her degrees in America, and accepted the arduous duties of the above excellent institution. From personal knowledge we may say that the Sheltering Arms is to be congratulated on having secured Dr. Abbey's services.

The following vacancies are announced this week, the amount of the salary in each case being given after the name of the institution:

**Resident Medical Officers.**

Derby Borough Asylum. Clinical Assistant—board, &c.
Bedford General Infirmary. Surgeon—£100, board, &c.
Northumberland County Asylum. Clinical Assistant—board, &c.
Royal Berks Hospital. House Surgeon—£80, board, &c.
Birmingham Skin and Lock Hospital. Third Officer.

**Non-Resident Medical Officers.**

Paddington Infirmary. Dispenser—£100.
German Hospital, Dalston. Hon. Medical Officer.

---

The only suitable New Year's gift this year has been a pocket-handkerchief; for we are all, more or less, the victims of influenza. How many of us waking with pain-racked limbs, and aching head, have imagined we were in for some fever, and, after all, it has turned out to be the unpleasant, but comparatively harmless influenza. Three days or a week of depression and discomfort, and, lo! we are ourselves again, and ready to laugh at our neighbours. Speaking from personal experience, a day in bed, low diet, and a blue pill, is the best treatment for the present epidemic; the only danger is the pneumonia and pleurisy which sometimes follow in its wake, but which can generally be avoided by a little care and common-sense.

---

On Christmas Eve, the Countess of Zetland, attended by Mr. John Mulhall, private secretary to the Lord Lieutenant, visited the Royal Hospital for Incurables, Donnybrook. Her Excellency was conducted through the hospital by Mr. Drummond, who pointed out the many additions and alterations being made in the old portion of the building—viz., new dining-hall, passenger lift, food lift, improved accommodation for the resident officials and for the nurses. All the wards were visited, and the countess handed Christmas cards to each of the patients, of whom there are present 169, all afflicted with incurable disorders, such as consumption, heart disease, paralysis, or that dread malady, cancer, and nearly all of whom have been previously treated in one or more of the general hospitals of Dublin, in which they could not be retained after their cases had been declared hopeless. The wards were tastefully decorated, and many pleasing mottoes and words of welcome were displayed.

---

The Empress Frederick while at Naples has visited the International Hospital, the history of which, as told by the *Times*, is very interesting. Formerly a small building admitting only Germans, it has expanded, and now welcomes the suffering of all nations and all creeds. Prof. Schrön is the man who has broken through the barrier erected by mistaken patriotism and narrow creeds, and thrown open the doors of the hospital to all sufferers from malady or accident. A small English hospital, which long struggled against many difficulties, has been merged in it, so that it is now truly international. An important feature in its management is that patients are permitted to call for the assistance of any medical man with whom they may have most sympathy. This is a most desirable arrangement in an institution which receives persons of every country and language, and is gratefully acknowledged by our English sailors especially. It is supported by contributions and annual subscriptions, and has an undoubted claim on the support of every Government whose subjects are assisted by it. Some years have passed since the late Lady Bentinck contributed to the funds of the hospital 150,000 lire, equal to £6,000, which enabled the committee to purchase the Villa Rendé. The Empress expressed herself greatly pleased with the organisation of this charity.

Many of the daily papers gave gruesome accounts of Christmas deaths—this man choked by plum pudding, that man killed by joy because the parish guardians gave him five shillings. But the most extraordinary tale comes from New York, where the friends of a lady provided a brass band to play for her as a pleasant surprise. On hearing the band the lady fell down and died. Many of us have felt inclined to do likewise when we have heard brass bands in London.

---

We have again to thank Madame Monchablon for dolls and useful articles sent for sick children, and made by the staff of type-writers employed by Madame Monchablon. Some of the dolls are very pretty, and some little quilts made of list will be most useful. Many thanks to those busy women who yet found time to work for the little children. All Madame's gifts were sent to the Hospital for Sick Children, Great Ormond-street. One of them, the magic lantern, was shown in all the wards on New Year's night, to the great delight of the children.

---

It may tend to allay the alarm which many editors seem bent upon exciting in regard to the Russian influenza epidemic, to publish the substance of a communication we have received from a gentleman in St. Petersburg. Not only does he not mention the epidemic of which the papers have been so full, but he states that, writing on Christmas Day (old style), the English colony are celebrating Christmas with much heartiness, and that they are looking forward to twelve days later, when they will celebrate Russian or double Christmas Day. He had received a plum pudding from England, which he and his friends were preparing to discuss with considerable heartiness and satisfaction.

---

The annual meeting of the Ayr Fever Hospital and Infirmary has been held. The financial statement showed a total income to the maintenance fund of £2,786 0s. 11d., including a balance from last year of £638 15s.; the expenditure amounted to £2,158 10s.; and there is a balance in bank of £255 2s. 1d.; £527 10s. was added to the capital fund, being one-half of special donations for the year, the other half going to the maintenance fund. The general subscriptions, exclusive of these special donations, amounted to £1,101 12s. 10d. The capital fund now amounts to £16,632 10s. The past year has been one of much activity in all departments, and a serious outbreak of fever has caused a large increase in the number of admissions to the institution. Four years ago the total number of cases treated was 469, while this year the number is 643.

---

Stinging children on the lips with stinging nettles is not exactly the mild and moral punishment we would expect from a lady superior. Unluckily, it has been proved before now that the religious habit can cover a hard heart, and that a sisterhood is not always that haven of peace and goodwill which outsiders imagine it to be. There are certain evil traits of the human character which are fostered by excessive discipline, and hardness of heart is one of them. Those who have no pity on themselves seldom have pity for others, and all the softening and beautiful influences of life being shut out of convent walls, the women within are likely to develop into mere machines. The enquiry into the Kilhampton Home shows how little sympathy was there felt for little children; it also shows what strict enquiries should be made concerning charitable "homes" before poor children are entrusted to their charge. The fact that the institution is charitable does not always mean that it is lovable; charity is taken in the narrower sense now than as used by St. Paul.

